# Supplementary material for: Optically controlling the competition between spin flips and intersite spin transfer in a Heusler half-metal on sub–100-fs time scales
Source: Sci Adv. 2023 Nov 10;9(45):eadi1428. doi: 10.1126/sciadv.adi1428 (PMC10637748; doi:10.1126/sciadv.adi1428)
Supplement: Supplementary file 1 — Figs. S1 to S8 [file sciadv.adi1428_sm.pdf]

Science Advances
AAAS

Supplementary Materials for

Optically controlling the competition between spin flips and intersite spin transfer in a Heusler half-metal on sub–100-fs time scales

Sinéad A. Ryan *et al.*

Corresponding author: Sinéad A. Ryan, sinead.ryan@colorado.edu

Sci. Adv. **9**, eadi1428 (2023)
DOI: 10.1126/sciadv.adi1428

This PDF file includes:

Figs. S1 to S8

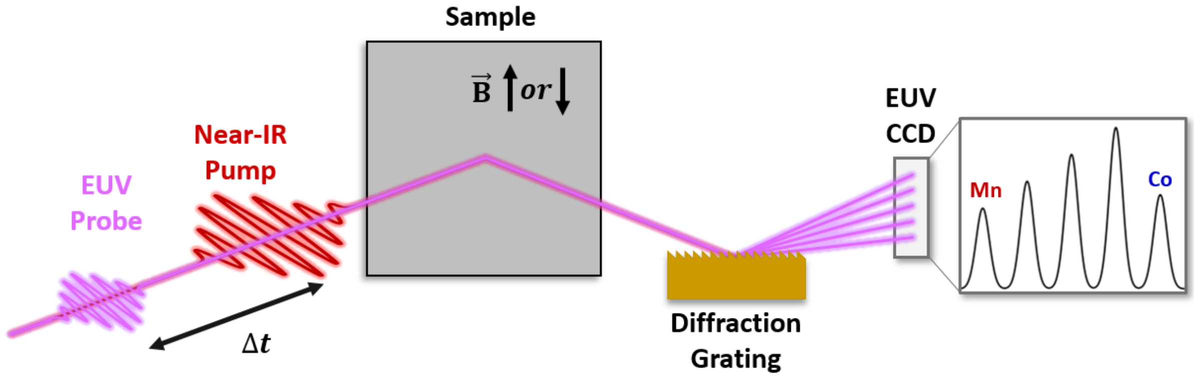

Fig. S1. Experimental design. The magnetized sample is excited with a near-IR pump pulse followed by an EUV probe pulse after a time delay Δt. The EUV probe contains a comb of energies produced by high harmonic generation. The harmonic comb is spectrally dispersed using a diffraction grating then detected with a CCD camera. Different harmonic energies are resonant with the M-edges of Mn and Co. TMOKE measurements are made by comparing the intensity of light reflected from the sample with two different sample magnetization directions (up and down) as shown in the figure.

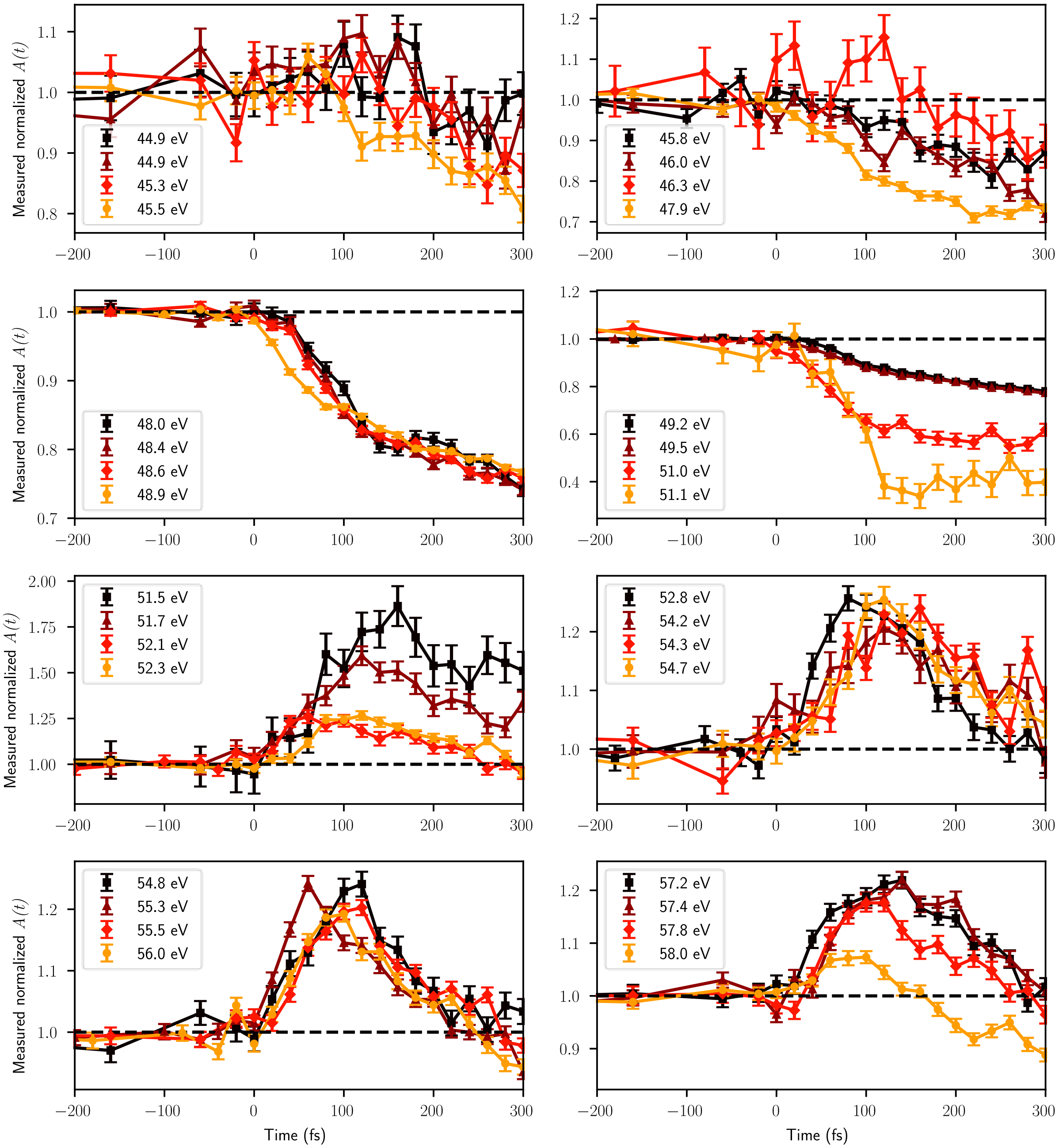

Fig. S2. Energy dependent transient magnetic asymmetry with probing energies from 44.9 eV to 58.0 eV. The peak of the Mn asymmetry occurs at 49.2 eV. The probing energy region from 51.5 eV to 55.0 eV lies between the Co and Mn edges. Signals at these probing energies see a combination of high energy Mn states as well as low energy Co states. We do not draw any conclusions from the enhancements seen in this region due to their strongly mixed nature.

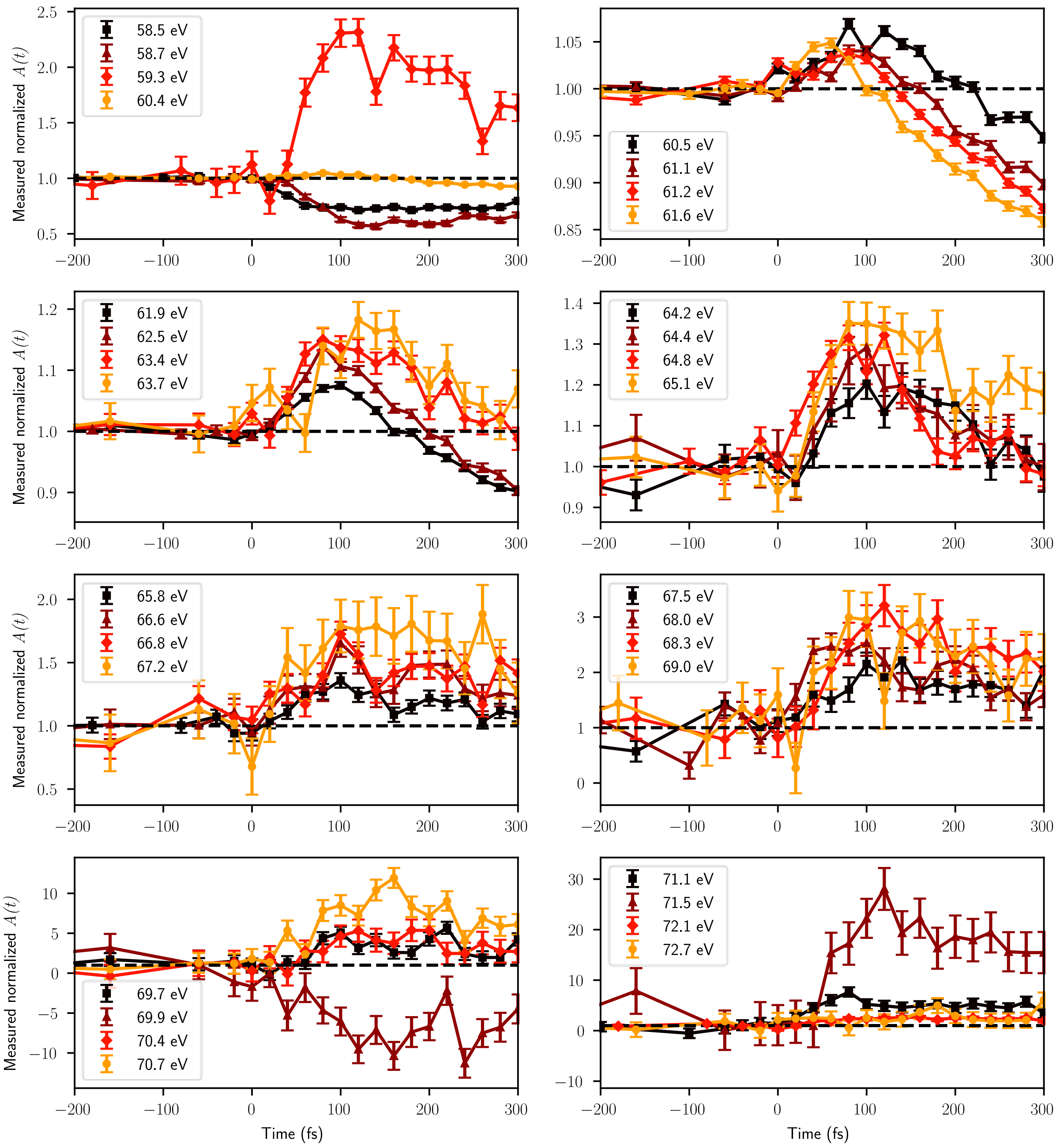

Fig. S3. Energy dependent transient magnetic asymmetry with probing energies from 58.5 eV to 72.7 eV. The peak of the Co asymmetry occurs at 60.4 eV.

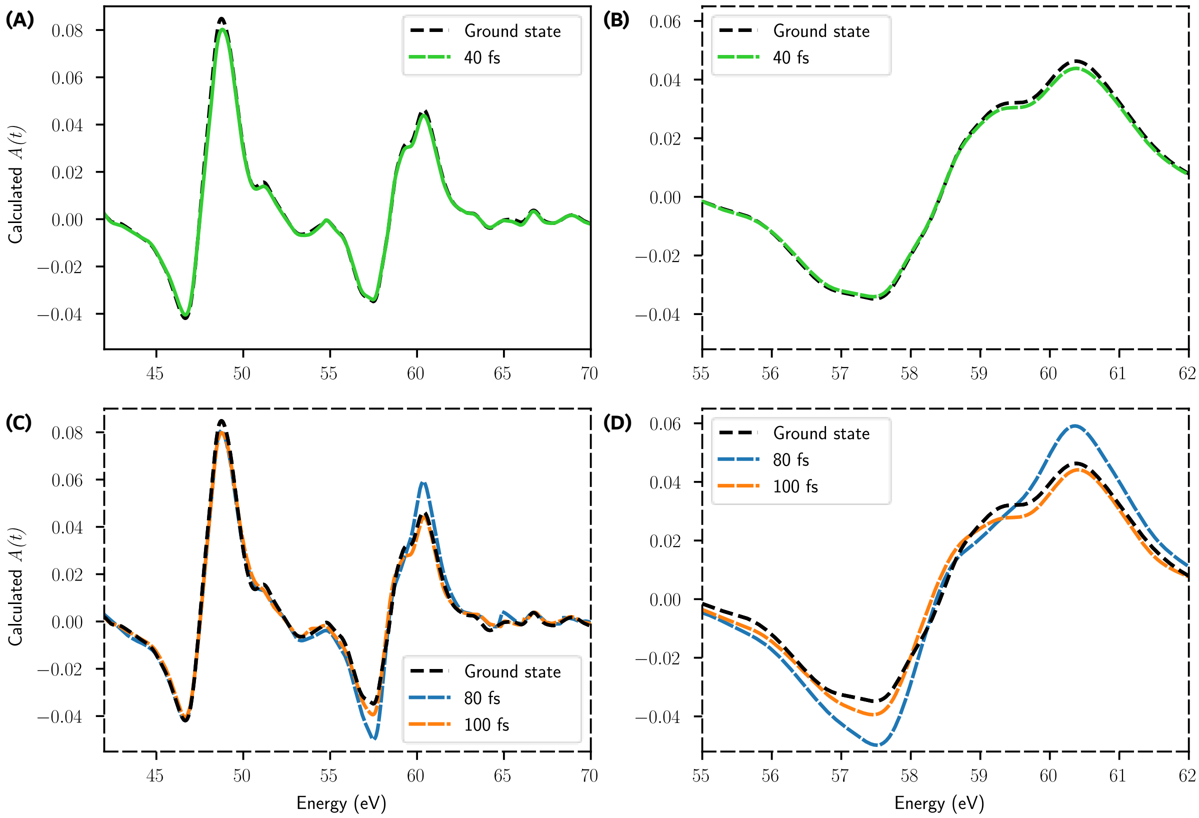

Fig. S4. Theoretical asymmetry curves at 0 fs, 40 fs, 80 fs and 100 fs following laser excitation from the pump pulse. (A) The asymmetry at 40 fs plotted across both the Co and Mn edges and (B) zoomed in on the Co-edge. At 40 fs, we see a reduction in asymmetry at the Mn and Co resonant peaks due to spin-flips. (C) The asymmetry at 80 fs and 100 fs and (B) zoomed in at the Co-edge. At 80 fs, we see enhancements across the Co-edge due to spin transfers. At 100 fs, the spin transfer excitations have mostly decayed and spin-flips begin to dominate once again.

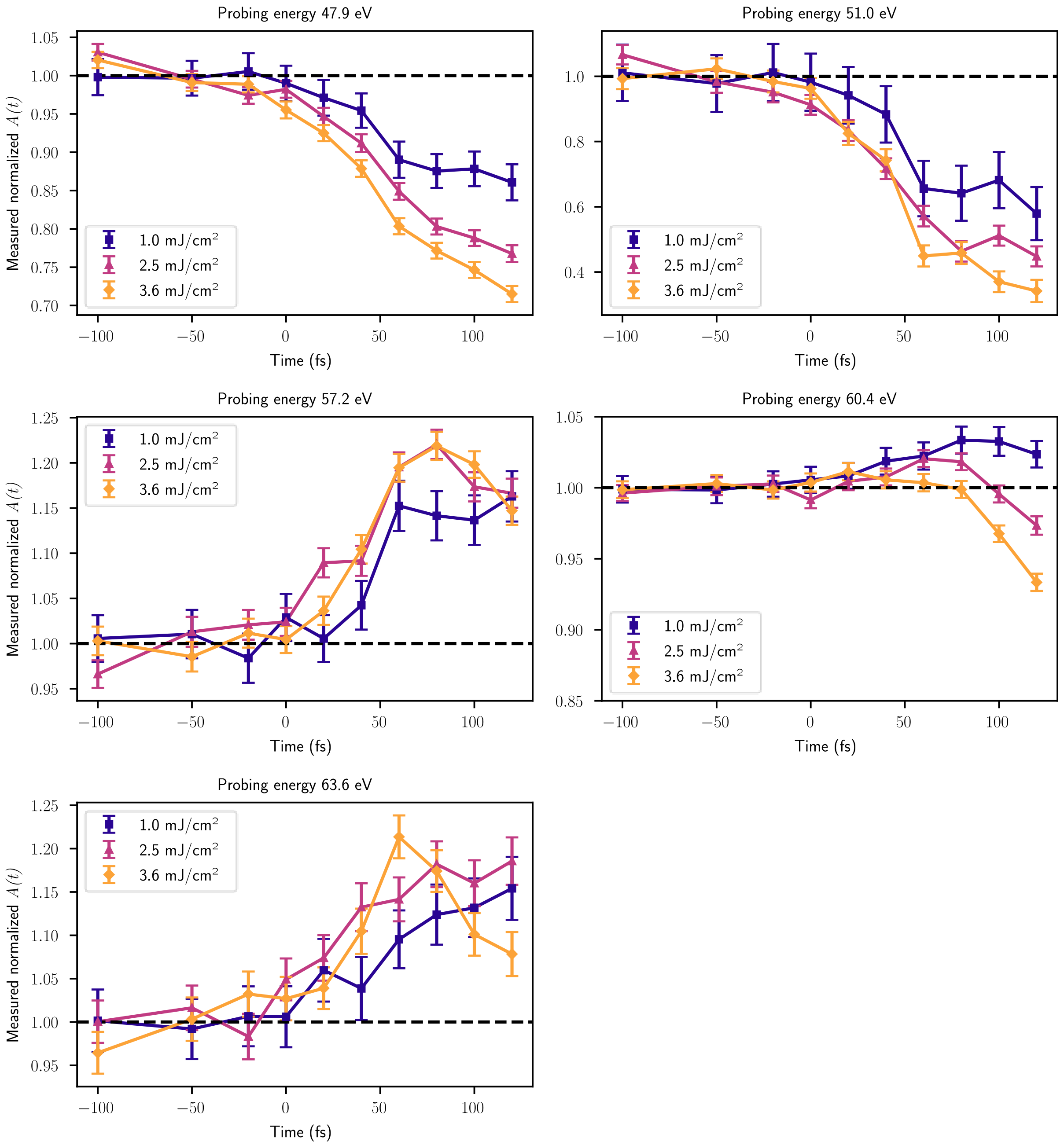

Fig. S5. Fluence dependent transient magnetic asymmetry measurements with five different probing energies from 47.9 eV to 63.6 eV. Around the Mn-edge, (i.e. 47.9 eV and 51.0 eV), demagnetization dominates at all fluences. Above and below the Co-edge at 63.6 eV and 57.2 eV, spin transfer dominates and the signal in enhanced. At the Co-edge, 60.4 eV, there is fluence dependent competition between spin transfer and ultrafast demagnetization.

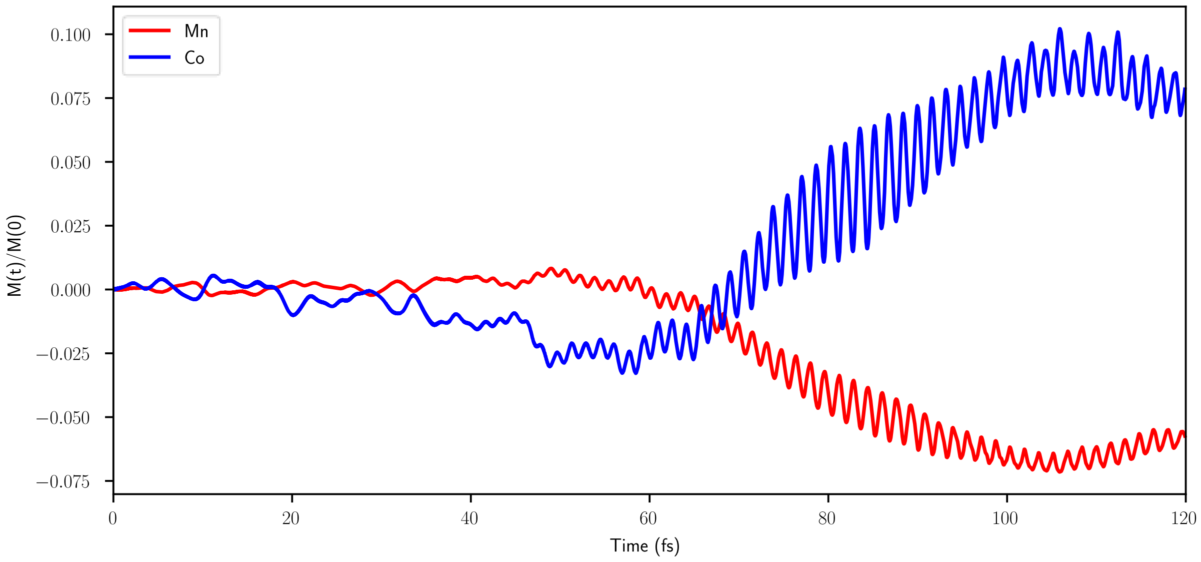

Fig. S6. The simulated transient changes in magnetic moment of Co and Mn with a pump fluence of 8.4mJ/cm^2. Here, a large calculated OISTR effect of 10% for Co is depicted for an 8.4 mJ/cm^2 pump fluence. In comparison, the Co moment only increases by ~2.5% for 2.2 mJ/cm^2 pumping. However, we note that the simulation does not include all effects that would contribute to demagnetization of Co such as: magnon generation, electron-phonon interactions, and superdiffusive spin currents. For this reason, we expect that this is an overestimation of the strength of the Co moment increase.

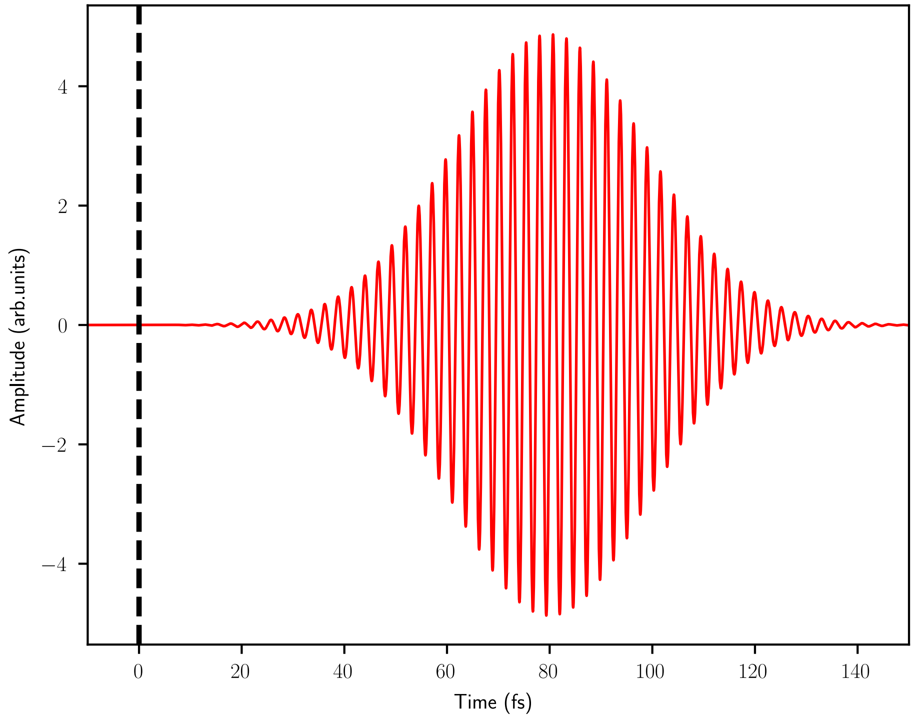

Fig. S7. The theoretical definition of *t*=0 relative to the time dependent amplitude of the simulated incident pump pulse. The theoretical time zero was chosen to maximize agreement with the experimentally determined time zero.

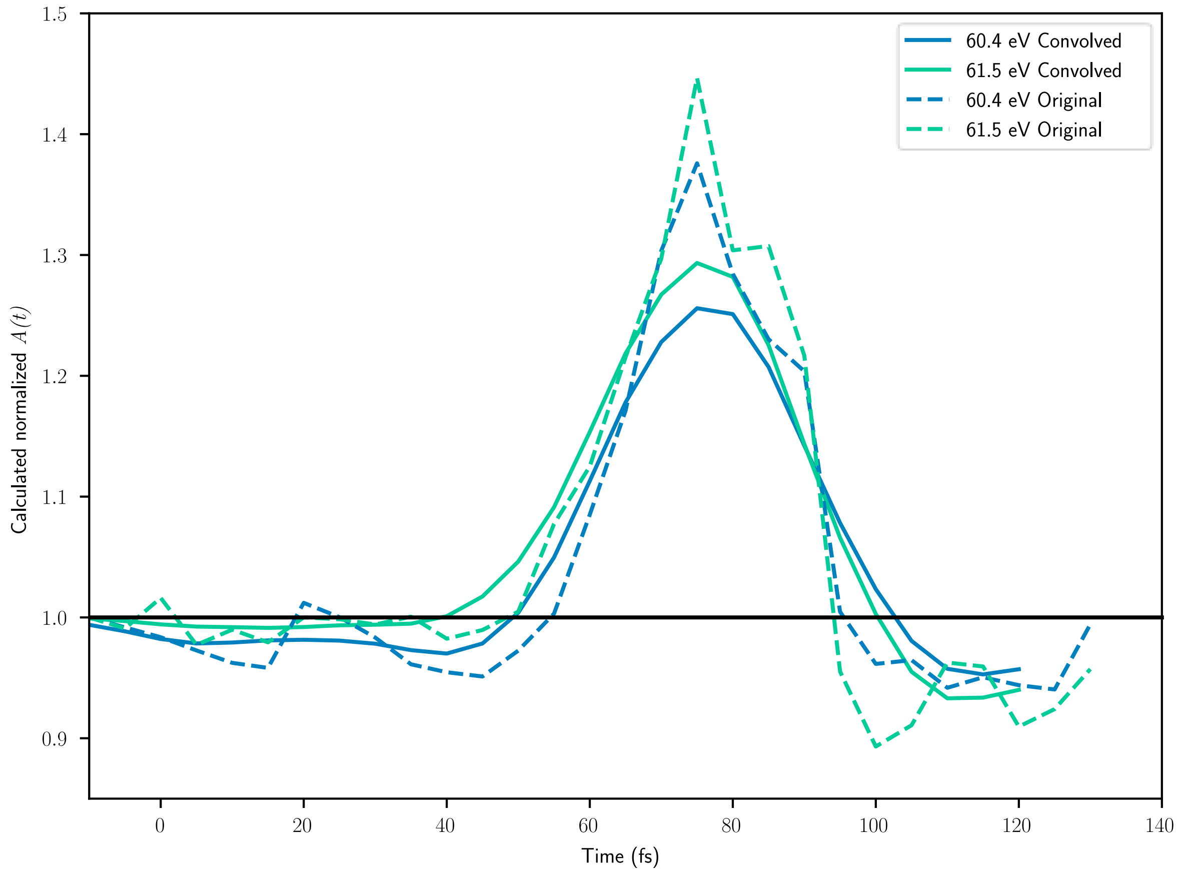

Fig. S8. The calculated theoretical magnetic asymmetry dynamics with and without convolving with a measurement probe. Dashed lines: the instantaneous magnetic asymmetry calculated for two different probing energies: 60.4 eV and 61.5 eV in steps of 5 fs. Solid lines: the same theoretical data convolved with a 25 fs FWHM probe pulse as described in the main text Fig. 2(D).
